# Detection of left atrial scar and changes of cardiac function in relation to AF ablation, by 3D late gadolinium enhancement

**DOI:** 10.1186/1532-429X-16-S1-P145

**Published:** 2014-01-16

**Authors:** Jose H Flores-Arredondo, Gerd Brunner, Lucien N Abboud, Joel D Morrisett, Christie M Ballantyne, Amish S Dave, William A Zoghbi, Miguel Valderrabano, Dipan J Shah

**Affiliations:** 1 Cardiology, Methodist Debakey Heart and Vascular Center, Houston, Texas, USA; 2 Section of Atherosclerosis and Vascular Medicine, Department of Medicine, Baylor College of Medicine, Houston, Texas, USA; 3 Internal Medicine, Baylor College of Medicine, Houston, Texas, USA

## Background

Atrial Fibrillation (AF) is the most common arrhythmia in clinical practice; catheter ablation (AFCA) is a widespread treatment. Determinants of AFCA success and post-AFCA left atrial remodeling are poorly understood. Delayed Enhancement Cardiac Magnetic Resonance (DE-CMR) has been used to image LA scar post-AFCA and its relation to ablation outcome. Here we explored the role of postablation LA scar in subsequent cardiac remodeling.

## Methods

We correlated post AFCA scar with cardiac volumes and function including LA volume, LV ejection fraction (LVEF), and LV cardiac output (LVCO). In a retrospective analysis, we identified 46 patients who underwent AFCA -pulmonary vein antral isolation in whom DE-CMR was obtained before and after AFCA (174 ± 160 days postablation). In 20 patients, a repeat AFCA procedure was performed due to recurrent AF. DE-CMR procedure was performed utilizing a navigated 3D inversion recovery gradient echo sequence (Siemens 1.5T Avanto or 3.0T Verio) approximately 15 minutes after administration of 0.2 mmol/kg Gadolinium (Magnevist). Scans were ECG-gated and acquired during a 150 ms window in mid-diastole with navigator-gating and fat suppression. We have developed an image analysis method and graphical user interface (GUI) to semi-automatically quantify hyperenhanced regions (scar) in the entire LA wall. Scar was quantified semi-automatically as fraction of LA contour in every slice from base to roof. STATA/IC 13.0 was used for statistical analysis. Two-way Interclass Correlation (ICC) for rater agreement was tested for LA perimeter and LA scar detection between two independent readers for 40 MRI slices. Variable normality was assessed with the Shapiro-Wilk test. The Wilcoxon signed-rank test was used to determine statistical significance between non-parametric variables in pre and post AFCA.

## Results

ICC indicates similarity between observers in both LA Perimeter (ICC: 0.785, CI: 0.59-0.886, p < 0.05) and LA Scar (ICC: 0.609, CI: -0.14-0.844, p < 0.05). The extent of LA Scar was higher in postablation (Median: 8.62, IQR: 5.43-16.4) DE-CMR compared to baseline (Median: 3.26, IQR: 0.55-8.3), with a p value < 0.001. LA volumes however were found to be lower in postablation (Median: 98.6 ml, IQR: 75.6-128.4) compared to baseline (Median: 109.5 ml, IQR: 90.3-136.1, p < 0.05) with a p value < 0.05. LVEF increased significantly (Pre Median: 60.69%, IQR: 54.91-66.67; Post Median: 64.8%, IQR: 60.05-68.68; p < 0.05) and LVCO increased (Pre Median: 5.00 L/min, IQR: 4.6-5.98, Post Median: 5.65 L/min, IQR: 5.01-6.68; p < 0.05), see Table.

## Conclusions

AFCA is associated not only with LA scar creation but also with significant favorable remodeling shown by decreases in LA volume and increases in LVEF and LVCO. The evaluation of scar and LA Perimeter through DE-CMR, using an in-home GUI is consistent among random readers.

## Funding

This work was supported in part by NIH grant T32HL07812.

**Table 1:** Comparison of Scar and Functional Values Pre and Post AFCA

Variable	**Mean ± Std. Dev**.	IQR Range	Prob > |z|
Pre LA Scar (%)	5.16 ± 5.66	0.55-8.3	0.0001*

Post LA Scar (%)	10.44 ± 6.98	5.43-16.4	

Pre LA Vol (ml)¥	121.622 ± 46.44	90.3-136.1	0.0006*

Post LA Vol (ml)	104.48 ± 38.05	75.6-128.4	

Pre LV C.O. (L./min.)	5.34 ± 1.36	4.6-5.98	0.0056*

Post LV C.O. (L./min.)	5.90 ± 1.29	5.01-6.68	

Pre LVEF (%)‡	59.52 ± 11.98	54.91-66.67	0.0152*

Post LVEF (%)	63.30 ± 8.37	60.05-68.68	

¥Higher in Persistent Afib vs. Paroxysmal. ‡ Lower in Persistent Afib vs. Paroxysmal. LA: Left Atrium. LA Vol: Left Atrial Volume. LA C.O.: Left Atrial Cardiac Output. LVEF: Left Ventricular Ejection Fraction. AFCA: Atrial Fibrillation Catheter Ablation.

**Figure 1:**
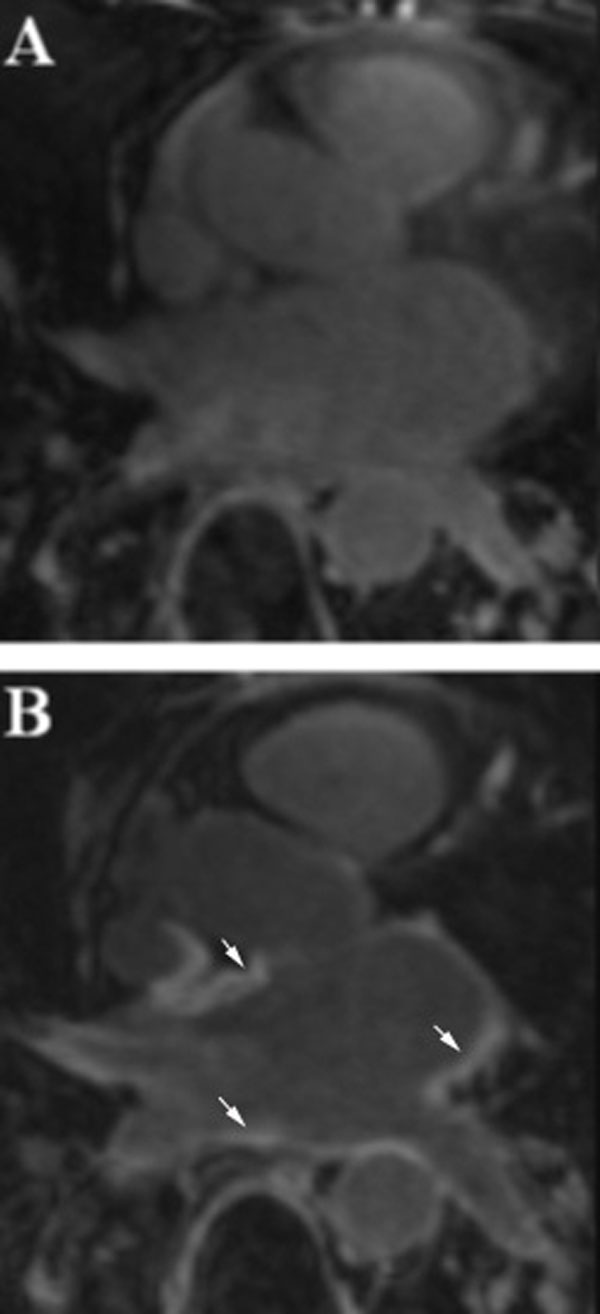
**Pre and Postablation DE-CMR images of the left atrium (same patient) A. Preablation Image (0.00% LA Scar, LA Vol. of 136.1 ml and LVEF of 60.69%)**. B. Postablation Image (16.24% LA Scar (arrows), LA Vol. of 76.9 ml and LVEF of 66.52%).

